# The Current Status of Mesenchymal Stromal Cells: Controversies, Unresolved Issues and Some Promising Solutions to Improve Their Therapeutic Efficacy

**DOI:** 10.3389/fcell.2021.650664

**Published:** 2021-03-16

**Authors:** David García-Bernal, Mariano García-Arranz, Rosa M. Yáñez, Rosario Hervás-Salcedo, Alfonso Cortés, María Fernández-García, Miriam Hernando-Rodríguez, Óscar Quintana-Bustamante, Juan A. Bueren, Damián García-Olmo, Jose M. Moraleda, José C. Segovia, Agustín G. Zapata

**Affiliations:** ^1^Hematopoietic Transplant and Cellular Therapy Unit, Medicine Department, Instituto Murciano de Investigación Biosanitaria Virgen de la Arrixaca, University of Murcia, Murcia, Spain; ^2^Spanish Network of Cell Therapy (TerCel), Instituto de Salud Carlos III, Madrid, Spain; ^3^Instituto de Investigación Sanitaria-Fundación Jiménez Díaz (IIS-FJD, Autonomous University of Madrid (UAM)), Madrid, Spain; ^4^Advanced Therapies Mixed Unit, Instituto de Investigación Sanitaria-Fundación Jiménez Díaz (IIS-FJD, Autonomous University of Madrid (UAM)), Madrid, Spain; ^5^Centre for Cytometry and Fluorescence Microscopy, Complutense University, Madrid, Spain; ^6^Hematopoietic Innovative Therapies Division, Centro de Investigaciones Energéticas, Medioambientales y Tecnológicas and Centro de Investigación Biomédica en Red de Enfermedades Raras, Madrid, Spain; ^7^Department of Cell Biology, Complutense University, Madrid, Spain

**Keywords:** MSC bioengineering, MSC homing, MSC immunomodulation, MSC preconditioning, MSC therapeutic efficacy

## Abstract

Mesenchymal stromal cells (MSCs) currently constitute the most frequently used cell type in advanced therapies with different purposes, most of which are related with inflammatory processes. Although the therapeutic efficacy of these cells has been clearly demonstrated in different disease animal models and in numerous human phase I/II clinical trials, only very few phase III trials using MSCs have demonstrated the expected potential therapeutic benefit. On the other hand, diverse controversial issues on the biology and clinical applications of MSCs, including their specific phenotype, the requirement of an inflammatory environment to induce immunosuppression, the relevance of the cell dose and their administration schedule, the cell delivery route (intravascular/systemic vs. local cell delivery), and the selected cell product (i.e., use of autologous vs. allogeneic MSCs, freshly cultured vs. frozen and thawed MSCs, MSCs vs. MSC-derived extracellular vesicles, etc.) persist. In the current review article, we have addressed these issues with special emphasis in the new approaches to improve the properties and functional capabilities of MSCs after distinct cell bioengineering strategies.

## Introduction

### Mesenchymal Stromal Cells: Lights and Shadows in the Knowledge of Their Mechanisms of Action

Numerous questions on the biology of mesenchymal stromal cells (MSCs), the most promising cell type for cell therapy strategies, remain unknown (Galipeau and Sensebe, 2018). This would explain the variability of both, the reported preclinical and clinical results and the difficulties to establish a general pattern of functioning for these cells. A current survey on the heterogeneity of MSCs, their immunogenicity, routes of delivery and migratory capacity, and principally on the mechanisms governing their immunomodulatory properties needs a substantial revision in order to design protocols for improving their therapeutic capacities, including MSC bioengineering.

MSCs were initially described as colony forming units-fibroblasts (CFU-Fb) capable of differentiating into distinct connective tissue lineages (i.e., osteoblasts, chondroblasts and adipocytes) (Friedenstein et al., 1970; Caplan, 1991; Pittenger et al., 1999). Multiple parameters can affect the therapeutic properties of MSCs including tissue origin (Ketterl et al., 2015), cryopreservation procedure (Oja et al., 2019), culture time and media supplementation with different growth factors (von Bahr et al., 2012; Moll et al., 2014b), optimal dosage (Golpanian et al., 2016) and *in vivo* cell delivery (Caplan et al., 2019; Moll et al., 2019) can affect substantially the cellular therapeutic properties of MSCs. Therefore, a better knowledge of these cell processes would improve the therapeutic outcomes of MSCs.

### Mesenchymal Stromal Cell Immunophenotype and Immunomodulatory Properties

There are no specific markers to characterize the immunophenotype of the MSCs. In humans, MSCs express CD73, CD90, CD105, CD166, CD29, and CD44 that are also present in many other cell types (Pittenger et al., 1999). Negative markers include CD34, CD45, CD14, CD11b, CD79a, CD10, and HLA-DR, except in the presence of IFNγ (Alfaro et al., 2020). In addition, they express numerous cytokine and chemokine receptors as well as distinct Toll-like receptors (TLRs) that play distinct immunomodulatory functions including the inhibition of T cell responses, antigen-presenting cell maturation, cytotoxicity of resting NK cells and differentiation of monocytes to immature dendritic cells (DCs) (Beyth et al., 2005; Jiang et al., 2005; Spaggiari et al., 2006; de Castro et al., 2019). Indeed, MSCs exhibit high plasticity over time and probably related with their origin in different microenvironments (Wilson et al., 2019). This MSC heterogeneity is due, at least in part, to the occurrence of distinct expression profiles (i.e., surface markers, transcriptome and proteome), and functional properties (Phinney et al., 2006; James et al., 2015; Mattar and Bieback, 2015). Some authors have proposed, but not conclusively demonstrated, that induced pluripotent stem cells (iPSCs)-derived MSCs could constitute a more homogeneous cell population (Bloor et al., 2020).

Nevertheless, it is important to clarify more conclusively the relevance of an inflammatory environment for the MSC-mediated immunomodulation. Two recent publications by Naserian and colleagues (Beldi et al., 2020a, b) have provided new and relevant information on the role played by TNF-α signaling in these processes. TNF-α exerts its effects through interaction with two receptors, TNFR1 and TNFR2. Whereas TNFR1 is ubiquitously expressed, TNFR2 expression is restricted to some cell types, including MSCs (Salomon et al., 2018; Yang et al., 2018). Remarkably, TNFR2 signaling results in pro-angiogenic and survival effects, but activation of TNFR1 signaling pathway generally induces apoptosis (Faustman and Davis, 2013). Furthermore, MSCs isolated from TNFR2 KO mice are less efficient in governing immunosuppression, including reduced capability to induce T cell differentiation toward Treg cell lineage (Beldi et al., 2020b). More recently, extended analysis of these TNFR2 deficient MSCs demonstrated that impeded TNFR2 signaling courses with reduced MSC colony-forming units (CFUs), proliferative rate and expression of diverse MSC cell markers. In addition, these deficient TNFR2 MSC produce more pro-inflammatory molecules (i.e., TNF-α, IFNγ, IL-6), less IL-10, TGFβ and nitric oxide (NO), and show reduced regenerative capabilities for wound healing, vascular tube formation and neoangiogenesis (Beldi et al., 2020a).

It has been proposed that the therapeutic properties of MSCs depend on the crosstalk of these cells with the host tissues (Ankrum et al., 2014; Galleu et al., 2017; de Witte et al., 2018; Galipeau and Sensebe, 2018), as suggested by the mechanisms which control their immunoregulatory properties and the status of pre-sensitization of host (Avivar-Valderas et al., 2019). During acute inflammation, MSC activation is critical for the production of immunoregulatory factors, in contrast with non-activated MSCs, which do not exhibit a significant production of these molecules. In acute inflammatory conditions, activated T cells secrete pro-inflammatory cytokines (i.e., IFNγ, TNF-α, IL-1, or IL-17), that activate MSCs initiating the modulation of immune responses by releasing anti-inflammatory molecules, such as prostaglandin E_2_ (PGE_2_), IL-10, HLA-G, indoleamine-2,3-dioxygenase (IDO), hepatocyte growth factor (HGF), TGFβ, NO, galectins, semaphorin-3A or heme-oxigenase (HO) as well as multiple chemokines (i.e., CXCL10, CXCL11, CXCL12, and CXCL19) (Jimenez-Puerta et al., 2020).

In general terms, activated MSCs in an inflammatory microenvironment block or largely inhibit activation of the complement system, neutrophils, T cells, B cells and NK cells. MSCs stimulate functional maturation of anti-inflammatory type 2 macrophages, regulatory DCs and B and T regulatory cells as well (Saparov et al., 2016; Wang et al., 2018, 2019; Jimenez-Puerta et al., 2020). Therefore, TLRs and numerous immunomodulatory factors secreted or expressed by MSCs are orchestrated to function together. Indeed, it has not been possible to identify one single mechanism responsible for the immunomodulatory properties of MSCs and distinct factors seem to act, coordinately and/or sequentially in the blockade of the immune system (Ferreira et al., 2018). On the other hand, PGE_2_ production largely depends on IL-10 signaling and MSCs stimulated by kinurenin through aryl-hydrocarbon receptors (AhR) show an increased production of iNOS, IDO and PGE_2_ (Jiang et al., 2005; Wang et al., 2018; de Castro et al., 2019). Moreover, low levels of TGFβ correlate with reduced IDO (Xu et al., 2014), and TNF-stimulated gene 6 (TSG6), that inhibit neutrophilia by blocking CXCL8-mediated chemotaxis, is regulated by AhR and IDO (Wang et al., 2018). Moreover, TLR2 activation induces galectin-3 production, increasing its capacity to suppress T cell activation (Sioud et al., 2010). However, effects mediated through TLR3 and TLR4 are controversial, although it is generally assumed that TLR3 signaling induces an anti-inflammatory MSC profile (MSC-2), while TLR4 signals promote pro-inflammatory MSCs (Shammaa et al., 2020). Importantly, and apart from their immunomodulatory properties, other studies have found that MSCs also possess robust anti-bacterial properties through secretion of a variety of anti-microbial peptides and/or proteins such as lipocalin-2, IL-37, hepcidin, keratinocyte growth factor and β-defensin-2 which has led to MSCs being considered as a therapeutic option for sepsis and septic shock (Krasnodembskaya et al., 2010; Gupta et al., 2012; Alcayaga-Miranda et al., 2015; Sung et al., 2016).

The key role played by Treg cells for governing MSC-mediated immunosuppression deserves further, more extensive analysis. MSCs induce Treg cell differentiation by increasing production of PGE_2_, TGFβ and IL-10. In addition, they increase Treg cell proliferation via TLR2 and TLR3 signaling, thrombospondin, IL-2 and TNF-α through activation of Stat5 that increases CD39 and CD73 expression, both molecules involved in the adenosine production necessary for Treg cell function (de Castro et al., 2019). On the other hand, as previously indicated, a close relationship has been established between TNFR2 expression and Treg cell function (Salomon et al., 2018; Yang et al., 2018; Naserian et al., 2020). Remarkably, Treg lymphocytes express TNFR2 which is directly related to their immunosuppressive effects (Leclerc et al., 2016; Naserian et al., 2020).

On the other hand, some reports suggest that systemically injected MSCs have immunosuppressive properties because they are entrapped in the lung microvasculature, die by apoptosis and are engulfed by local macrophages that become type 2 macrophages which secrete IL-10 and arginase immunosuppressive factors (Anderson et al., 2013; Braza et al., 2016). Engulfed MSCs appear mainly in non-classical Ly6C^low^ monocytes that polarize toward CD14^+^CD16^+^CD296^+^ monocytes, an intermediate phenotype with anti-inflammatory properties that produces IL-10 and express PDL-1. In addition, these primed monocytes that engulfed MSCs induce CD4^+^CD25^high^ Treg cell formation (Weiss and Dahlke, 2019).

These results indirectly support that apoptotic, metabolically inert or even fragmented MSCs would have the same immunomodulatory properties as living MSCs (Chang et al., 2012; Galleu et al., 2017; Weiss and Dahlke, 2019). Therefore, the viability of MSCs would not be a pre-requisite for some of their exerted immunomodulatory effects (Weiss and Dahlke, 2019). In this respect, Thum and colleagues pointed out that the apoptosis of MSCs is caused by modulation of both innate and adaptive immunity (Thum et al., 2005) and further studies support this idea. Apoptotic MSCs exhibited an immunosuppressive behavior in a Th2-type inflammatory model, inducing IDO production in host phagocytic cells (Galleu et al., 2017), and supernatants of cultured macrophages that engulfed MSCs improved the survival of hypoxic cardiomyocytes (Lu et al., 2013). Remarkably, systemic administration of apoptotic adipose tissue-derived MSCs provide better therapeutic results than the treatment with living MSCs in a cecum ligation and puncture-induced sepsis model (Sung et al., 2013). On the other hand, MSCs heated for 30 min at 50°C that provokes an irreversible blockade of cell metabolism but maintains the cell integrity, were able to reduce the inflammatory response in mice receiving LPS by a significant reduction of the serum levels of IFNγ and increased production of IL-10 (Luk et al., 2016). Also, normal MSCs and metabolically inactive MSCs showed similar effects on monocyte function with a significant reduction of TNF-α production in response to LPS (Jiang et al., 2005). By contrast, the intrapulmonary administration of apoptotic MSCs did not increased survival or reduced the severity of endotoxin-induced acute lung injury (Gupta et al., 2007), in contrast with several studies demonstrating the significant positive effect of living MSCs in the reduction of sepsis in different experimental models (Gupta et al., 2007; Johnson et al., 2017).

## Mesenchymal Stromal Cell Manufacturing

MSC manufacturing for clinical use has been regulated worldwide for over a decade in an attempt of protecting potential users. Production of cell medicaments with protocols accepted by regulatory agencies under GMP conditions generates a cellular product with specific properties and a high level of safety. Autologous MSC manufacture was the first step and these cells are currently used in the majority of clinical trials and treatments; however, this procedure has disadvantages such as the time required to obtain an adequate number of cells from older or fragile patients, or the difficulty of growing MSCs *in vitro* from patients with different pathologies. For this reason, cryopreservation of cells has been frequently used to allow delayed treatment or for allogeneic donors; although cryopreservation is not an innocuous process for cells.

Cryopreservation has interesting benefits in clinical practices and is mandatory for MSC banking, but its effects on MSC biology are controversial. While some authors have discussed that the cryopreservation process reduces MSC potency, other studies have found no significant influence on their immunomodulatory properties (Cruz et al., 2015; Luetzkendorf et al., 2015). Two freezing steps with, at least, one preceding cell culture passage before freezing do not seem to affect the essential biological parameters of MSCs (i.e., cell yield, growth kinetics and population doubling number), but ≥4 freezing steps could accelerate the senescence of cultured MSCs. In addition, the immunosuppressive potential of frozen and thawed MSCs, independently of the number of freezing steps, is reduced by about 50% as compared to freshly cultured MSCs, but definitively do not abolish the process, even after long periods (>10 months) of cryopreservation (Klinker et al., 2017; Oja et al., 2019; Giri and Galipeau, 2020). Moreover, a variety of methods for long-term storage of MSCs using different formulations of cryopreservation media or different procedures for MSC freezing and thawing may subsequently greatly affect the MSC potency (Ding et al., 2010; Liu et al., 2010; Moll et al., 2014a; Miyagi-Shiohira et al., 2015; Rogulska et al., 2019). Therefore, the improvement of the cryopreservation conditions to ensure the intrinsic biological properties of MSCs needs further investigation in order to extend the utility of MSC banking for subsequent cell therapy uses.

On the other hand, the production of a sufficient number of MSCs by *in vitro* expansion for obtaining a clinical dose may have some impact on the native properties of the MSCs. Although MSCs can be growth up to 20 passages, these long-term cultured MSCs have shown senescence genes up-regulation, morphologic changes, decreased differentiation potential, chemokine receptor down-regulation, telomere length shortening and decreased immunosuppressive properties compared to short-term cultured counterparts (Jiang et al., 2002; Rombouts and Ploemacher, 2003; Honczarenko et al., 2006; Izadpanah et al., 2008; Li et al., 2012; Lian et al., 2016). Accordingly, the establishment of universal protocols for maintenance, banking and culture of MSCs would be welcome.

## Allogeneic or Autologous Mesenchymal Stromal Cells for Therapeutic Usage

A second controversial issue is whether allogeneic better than autologous MSCs would be used clinically. Indeed, both preclinical studies and clinical trials show an increasing use of allogeneic MSCs. Autologous MSC transplantation has some limitations. Firstly, the high cost of cell preparation just for a single recipient. Moreover, it is difficult to obtain a clinical dose of MSCs from some patients. For example, MSCs isolated from elder donors have decreased proliferation, less differentiation, and less regenerative potential, subsequently leading to ineffective treatments (Maredziak et al., 2016). By contrast, it is evident that the use of allogeneic vs. autologous MSCs for cell therapy has clear advantages (Hare et al., 2012; Zhang et al., 2015). Allogeneic MSCs from young healthy donors are an optimal choice to solve this problem. In addition, the expansion of autologous MSCs to obtain a clinical dose is time-dependent, making this therapeutic approach difficult for the early treatment of diseases in acute phase (e.g., COVID-19, brain stroke, septic shock or myocardial infarction). However, allogeneic MSCs, cryopreserved and stored once obtained, can be readily available, quickly thawed, and immediately administered to the patient who requires them. For all these reasons, cryopreserved allogeneic MSCs are a promising therapeutic alternative to autologous MSCs with multiple advantages in terms of time, cost of production and quality assurance. Importantly, allogeneic MSCs from pooled mononuclear cells of multiple third-party donors have been reported to exhibit decreased heterogeneity and to exert significantly higher immunosuppresive potential than those obtained from individual donors (Kuci et al., 2016).

However, this proposal leads to the unsolved question of the MSC immunogenicity. It is well known that there is immune activation of host cytotoxicity mediated by complement, NK cells and/or cytotoxic T cells (Noone et al., 2013; Ankrum et al., 2014; Berglund et al., 2017; Kot et al., 2019). In fact, syngeneic MSCs persist for more than 200 days, whereas allogeneic cells rapidly disappear (Eliopoulos et al., 2005). Although, low or null immunogenicity for allogeneic MSCs has been claimed (Le Blanc et al., 2003; Escacena et al., 2015), recent *in vivo* and *in vitro* evidence suggests that MSCs generate both innate and adaptive host immune responses (Caplan et al., 2019). However, anti-MSC responses are lower than those against other allogeneic cells are (Khan and Newsome, 2019), perhaps because MSCs do not express MHC class II antigens or co-stimulatory molecules. Therefore, the balance between their immunogenicity and the release of immunosuppressive factors, highly dependent on the local microenvironment, determines the MSC behavior (Khan and Newsome, 2019). Even more, this cytotoxic activity is important for MSC-mediated immunosuppression because it results in phagocytosis of apoptotic cells and then macrophage polarization (Galleu et al., 2017; de Witte et al., 2018). Thus, reduction of the activity of host immune system could diminish the efficiency of MSCs (Caplan et al., 2019).

Accordingly, the study of HLA matching between donor MSCs and recipient of these cells is being recently proposed (Avivar-Valderas et al., 2019). On a phase III clinical trial for the treatment of complex perianal fistulous pathology in patients with Crohn’s disease, the authors carried out a study on the immunological responses and MSC efficacy taking into account the haplotypes of the donor cells and the recipient concluding that an HLA-screening to the donor MSCs would be performed to limit the humoral response between donor and recipient.

## Delivery of Mesenchymal Stromal Cells

There is no consensus on the best method for MSC delivery (Caplan et al., 2019). Intramuscular delivery is a safe and simple method, but its efficiency is frequently low (Jahromi et al., 2019). In some organs, *in situ* direct injection is almost mandatory but may impede interactions between MSCs and host cells, particularly in lungs and spleen, thus limiting their therapeutic activity. In addition, delivery of a high number of cells could induce important cell damage, including high cell mortality by trauma, hypoxia or NK cell-mediated MSC apoptosis. On the contrary, systemic infusion of MSCs allows interactions with host cells and tissues but needs an adequate biodistribution and homing to affected tissues, which is sometimes limited. Intra-arteriolar delivery would be the most efficient method but also can be potentially harmful because MSCs mechanically entrap in the microvasculature elsewhere (Toma et al., 2009). The most frequently used method is the systemic delivery by intravenous injection but, particularly in rodents and in lesser extent in humans, results in a high number of entrapped MSCs in lung capillaries that limit the number of cells reaching target organs and increase the risk of thromboembolism (Scarfe et al., 2018; Coppin et al., 2019). Although there are only a few clinical trials reporting MSC-associated thrombotic events (Jung et al., 2013; Wu et al., 2017), MSC delivery triggers the activation of the complement system and the coagulation cascade inducing the so called “Instant Blood-Mediated Inflammatory Reaction” (IBMIR) (Moll et al., 2012, 2019). MSCs express the pro-coagulant tissue factor (CD142) (Drake et al., 1989), and MSC systemic injection significantly increases C3a and sC5b-9 levels and activation of the thrombin-anti-thrombin complex, inducing a drop in platelet numbers and increased values of D-dimer (Moll et al., 2015). These results remark the relevance of monitoring MSC pro-coagulant activity after their systemic infusion (Caplan et al., 2019). On the other hand, release of complement activation factors after exposure to MSCs could modulate their immunomodulatory and chemotactic activity (Schraufstatter et al., 2009; Moll et al., 2011), and protocols to avoid or attenuate complement-mediated cell damage would improve the efficiency of MSC-based therapies (Moll et al., 2016).

## Mesenchymal Stromal Cell Homing

Similar to leukocytes and hematopoietic stem cells, MSCs must undergo a multistep process to extravasate from the circulating blood and migrate through the vessel walls to the damaged tissues. This process includes various sequential steps: (1) an initial decelerative tethering followed by direct rolling contacts with endothelial cells; (2) activation of integrins (mainly induced by chemokines); (3) integrin-dependent firm adhesion to endothelial cells; (4) transendothelial migration; and (5) interstitial migration toward the injured tissue (Nitzsche et al., 2017). However, MSC homing to the damaged organs is very inefficient, and only a small proportion of cells reach target tissues (Devine et al., 2003). A restricted repertoire of functional homing and chemokine receptors exhibited by MSCs could be reason for this inefficiency (Honczarenko et al., 2006; Chamberlain et al., 2008). Among them, MSCs express neither the sialofucosylated glycoforms of CD44 nor P-selectin glycoprotein ligand-1 (PSGL-1). These molecules, called hematopoietic cell E-/L-selectin ligand (HCELL) and cutaneous lymphocyte antigen (CLA), respectively, contain the sialyl Lewis X (sLeX) moiety that mediate migration to E-selectin-bearing endothelial beds in sites of inflammation (Sackstein et al., 2008; Sackstein, 2009). In addition, the response of MSCs to CXCL12 gradients is controversial because it has been reported that they do not express its receptor, CXCR4 (Ullah et al., 2019). By contrast, MSCs extravasation is mediated by the expression of FGF receptors that interact with bFGF on endothelial cells mediating galectin-1-dependent adhesion to P-selectin (Langer et al., 2009). Then, MSCs send out filopodia and cross the intraluminal space with the concourse of metalloproteinases and the development of a front cell pole through their intracellular adaptor FROUNT and the chemokine receptor CCR2 (Zachar et al., 2016). But this mechanism of extravasation is less effective.

On the other hand, it has been found that *in vitro* prolonged expansion of MSCs in culture produces a down-regulation of a variety of homing molecules including chemokine receptors, such as CCR1, CCR7, CCR9, CXCR5, and CXCR6, thus lacking the chemotactic response to these chemokines (Rombouts and Ploemacher, 2003; Honczarenko et al., 2006). Accordingly, attempts for improving MSC homing are complex and require further optimization. Some of them have focused on introducing modifications in the expression of different homing molecules on migrating MSCs through a wide variety of genetic, enzymatic or ligand conjugation approaches. Enzymatic treatment of MSCs by α(1,3)-exofucosylation of the CD44 receptor with either stereospecific fucosyltransferase VI or fucosyltransferase VII in presence of its substrate GDP-fucose, or by fucosyltransferase VI gene transfection, has been shown to engender the potent E-selectin ligand HCELL on the MSC surface. This transient modification increases efficiently the *in vivo* tethering and rolling contacts on E-selectin-expressing endothelial beds in bone marrow microvasculature and in inflamed tissues (Sackstein et al., 2008; Abdi et al., 2015; Dykstra et al., 2016; Chou et al., 2017). Remarkably, recent findings have shown that exofucosylated MSCs display an altered secretome characterized by an augmented expression of anti-inflammatory molecules, leading to higher MSC immunosuppressive properties, as well as increased migration ability toward some pro-inflammatory chemokines such as CCL5, CCL20 and CXCL16 (Garcia-Bernal et al., 2020). Other authors reported that covalent binding of sLeX to the MSC surface through a biotin-streptavidin bridge, by conjugation of E-selectin-targeting peptide on the MSC membrane or by mRNA transfection to overexpress PSGL-1 and sLeX on MSCs resulted in an augmented rolling behavior on P- and E-selectin-coated surfaces and on inflamed vascular endothelium *in vivo* (Sarkar et al., 2008; Cheng et al., 2012; Levy et al., 2013). Lo et al. fused the first 19 aminoacids of PSGL-1 to human IgG and, after overexpression of this construct on HEK293T cells (a cell line with endogenous fucosyltransferase VII expression), they coupled this fusion protein to the MSC surface using palmitated protein G (PPG), leading to an increased rolling on P- and E-selectin-coated surfaces under hydrodynamic flow (Lo et al., 2013). By contrast, Ko et al. coated MSCs with PPG and anti-ICAM-1 antibodies, thus improving its ability to adhere to this endothelial ligand (Ko et al., 2009).

Engineering approaches for improving other MSC functional capacities, that will be described below, have been used for increasing MSC homing. Genetic modification by mRNA transfection is highly efficient and non-toxic to MSC as well as compatible with ectopic co-expression of multiple mRNAs at the same time (Kormann et al., 2011; Hamann et al., 2019). Using these types of strategies, Liao et al. tested the therapeutic capacity of engineered MSCs expressing PSGL-1, sLeX, and IL-10 via mRNA transfection in a mouse model of experimental autoimmune encephalomyelitis observing a decreased infiltration of immunocompetent cells into the white matter of the spinal cord (Liao et al., 2016). More recently, Hervás-Salcedo et al. have shown the improved therapeutic efficacy of human AdMSCs transfected with mRNAs encoding for specific migration and anti-inflammatory molecules. In particular, these data demonstrated that the transient co-expression of CXCR4 and IL-10 in human AdMSCs, using a single bicistronic mRNA, increases the migration of these cells to inflamed sites and enhances their anti-inflammatory properties in a local inflammation mouse model (Hervas-Salcedo et al., 2021).

Other experimental approaches were focused on the overexpression of the chemokine receptor CXCR4 for enhancing migration and mobilization of MSCs through activation of the CXCL12/CXCR4 signaling pathway. As above indicated, CXCR4 is usually absent on the surface of culture-expanded MSCs, but after the *in vitro* treatment of MSCs with diverse cytokines it is highly expressed (Rombouts and Ploemacher, 2003; Shi et al., 2007). Thus, pre-treated MSCs with insulin-like growth factor 1 (IGF-1) for 48 h markedly increased the CXCR4 expression *in vitro*, and a greater number of MSCs treated with IGF-1 engrafted and survived in the peri-infarcted area when the cells were transplanted in a rat model of myocardial infarction (Guo et al., 2008). IL-3-pre-conditioned human MSCs up-regulated the CXCR4 expression, enhancing their *in vitro* migration toward CXCL12 and their *in vivo* migration in immunocompromised mice (Barhanpurkar-Naik et al., 2017).

Another strategy to increase the CXCR4 expression in MSCs is by genetic modification. Zheng et al. (2019) transduced mouse bone marrow derived-MSCs with a lentiviral vector carrying the CXCR4 gene. Then, mice suffering colitis associated tumorigenesis, injected with MSCs-CXCR4 showed relieved weight loss, longer colons, lower tumor numbers and decreased tumor burden compared to mice receiving the unmodified MSCs. Kim et al. (2017) demonstrated in a mouse diabetic hindlimb ischemia model that CXCR4-overexpressing adipose derived-MSCs contributed more efficiently to the early homing and engraftment into ischemic areas than unmodified MSCs, also improving the long-term engraftment and muscle tissue regeneration.

Other strategies aimed to improve the homing capacity to target tissues include the employment of different scaffolds (i.e., hydrogels and chitosans) (Schantz et al., 2007; Shen et al., 2010; Thevenot et al., 2010), magnetic guidance after MSC labeling with iron oxide magnetic particles (Arbab et al., 2004; Yanai et al., 2012; Yun et al., 2018), coated MSCs with biotinylated lipid vesicles, and irradiation or pulsed-focused high intensity ultrasounds that frequently improve MSC engraftment by up-regulating CXCL12 release by activation of different mechanotransduction pathways (Ziadloo et al., 2012; Zang et al., 2017; Liu et al., 2020). Nevertheless, these are complex methods that require a rigorous optimization (Ullah et al., 2019).

## Engineering Mesenchymal Stromal Cells for Enhancing Their Therapeutical Properties

The myriad of processes that governs the biology and function of MSCs makes difficult to manipulate them for improving their therapeutic possibilities. Different experimental approaches have engineered MSCs (i.e., MSCs-2.0) aimed to enhance their therapeutic efficacy compared to native MSCs and have been tested in several preclinical models of a variety of diseases. MSCs have been mainly modified to increase their survival, retention, migration capacities and growth factor production, principally through genetic modifications, usually achieved by means of viral vectors but also using non-viral methods. Standard protocols can reach high levels of transduction without affecting the lineage differentiation or the intrinsic properties of MSCs. Constitutive rather transient transformation provides the best therapeutic effects (Lin et al., 2011). The most common vectors used to modify MSCs are retrovirus, lentivirus, adenovirus and adeno-associated virus (AAV) (Sage et al., 2016). Among the non-viral approaches mRNA lipofectamine-mediated transfection, PEGylated DNA template nanocomposite system, biotinylated MSC, spermin pullulan, hyper-branched polyamidoamine and jetPEI-mediated transfection have been used (Pawitan et al., 2020).

On the other hand, several studies described that the incorporation of anti-inflammatory genes such as IL-10, HGF, IDO and FoxP3 could improve the therapeutic potential of MSCs. The overexpression of other factors including VEGF, BMP2, osteogenic molecules (i.e., TGFβ, Cbfa-1, and Osterix), or molecules involved in homing (CXCR4 and CXCL12), etc. have been shown to enhance the MSC capacities (Pawitan et al., 2020). Particularly, enhanced IL-10 production has been intensively tested. IL-10 is a strong anti-inflammatory cytokine produced by monocytes/macrophages, Th2 lymphocytes and regulatory T cells. IL-10 inhibits the production of pro-inflammatory cytokines by Th1 lymphocytes and improves survival, proliferation and antibody production of B-lymphocytes. Therefore, enhanced IL-10 expression could represent a promising therapeutic approach for diverse pathologies in which immunosuppression is needed (Grutz, 2005; Mosser and Zhang, 2008).

As previously indicated, triple-transfected PSGL-1/sLeX/IL-10 MSCs injected in an mouse model of local inflammation in the ear, induced a transient increase in the levels of IL-10 in the inflamed ear, and mediated a superior anti-inflammatory effect *in vivo* compared to *wild type* MSCs (Levy et al., 2013). These results are also supported by the cited above study in which the authors demonstrated the enhanced anti-inflammatory potential of human AdMSCs transfected with a single mRNA encoding for the receptor CXCR4 and IL-10 (Hervas-Salcedo et al., 2021).

The administration of IL-10-transduced bone marrow allogeneic MSCs attenuated the severity of acute graft-vs.-host disease in a murine model, while unmodified MSCs were not able to control the disease progression (Min et al., 2007). Different studies found that serum levels of IL-10 in rheumatoid arthritis-suffering patients was lower than that found in healthy people, but some pro-inflammatory factors, such as IL-17, IL-1β and TNF-α, were higher (Baek et al., 2012; Shoda et al., 2017). Using an adenovirus system to overexpress IL-10, Tian et al. analyzed the therapeutic effect of IL-10-overexpressing bone marrow-derived MSCs (IL10-BMMSCs) in a collagen-induced rheumatoid arthritis rat model. After 4 and 8 weeks of treatment IL-10-BMMSCs receiving rats improved significantly their clinical condition. The repairing rate of osteoarticular cartilage and the inhibition of synovial proliferation were higher in the IL-10-BMMSCs group than in the unmodified counterparts. Accordingly, serum levels of the pro-inflammatory cytokines IL-17, IL-1β, and TNF-α were also lower (Tian et al., 2019). In a model of *Escherichia coli* pneumosepsis in rats, IL-10 overexpression in umbilical cord derived-MSCs (UC-MSCs) enhanced the capacity to attenuate lung injury compared to unmodified UC-MSCs, due to increased macrophage phagocytosis and killing of *E. coli* (Jerkic et al., 2019). Recently, Zhao et al. also found that MSCs transfected with a recombinant plasmid IL10-PEGFP-C1 were able to suppress the pancreatic cancer cell proliferation *in vitro* and to reduce the growth of tumor xenograft *in vivo*, prolonging the mouse survival, inhibiting tumor angiogenesis and reducing blood levels of TNF-α and IL-6 in mice with tumors (Zhao et al., 2020).

IL-10 has been also claimed to play a neuroprotective and vasculoprotective role in cerebrovascular disorders by attenuating pro-inflammatory signals and by upregulating anti-apoptotic proteins (Zhou et al., 2009). Nakajima et al. investigated the therapeutic benefit of adeno-associated virus (AAV)-mediated IL-10 overexpression in MSCs transplanted during the acute phase of ischemic stroke in Sprague-Dawley rats. MSC-IL10 grafting significantly inhibited microglial activation and pro-inflammatory cytokine expression. Moreover, overexpression of IL-10 suppressed neuronal degeneration and improved survival of engrafted MSCs in the ischemic hemisphere (Nakajima et al., 2017).

In a rat model of myocardial infarction, Meng et al. transduced bone marrow-derived MSCs using an adenoviral vector to secrete IL-10 (Ad.IL-10−MSCs). These modified MSCs were transplanted into injured hearts resulting in reduced myocardial infarcted area, cardiac impairment and cell apoptosis. Even, genome-editing technology using transcription activator-like effector nucleases (TALENs) has been used to generate functionally improved amniotic MSCs (Meng et al., 2019). The administration of these IL-10 gene-edited amniotic MSCs in an acute myocardial infarction mouse model showed higher anti-inflammatory properties and enhanced recovery of heart function, also providing a favorable environment for neovascularization.

On the other hand, FoxP3-expressing MSCs prevent rejection of allogeneic grafted liver, increasing the median survival time of treated mice by increasing the numbers of Treg cells and the PD-L1 expression on MSCs (Qi et al., 2015). In other studies, HGF-expressing MSCs exhibited enhanced regenerative and anti-apoptotic effects in murine models of radiation-induced toxicity (Zhang J. et al., 2014; Wang et al., 2015), and induction of early immunosuppression in mice undergoing rheumatoid arthritis (Dong et al., 2020). Bcl-2 is also a robust anti-apoptotic protein, which has been overexpressed in MSCs. These cells ameliorated myocardial infarction damage in mice by increasing cell engraftment and VEGF-mediated neovascular formation (Li et al., 2007). Other factors overproduced by MSCs (i.e., lipocalin-2) indirectly improved their therapeutic capacity inducing production of regenerative factors such as HGF, IGF, FGF, and VEGF (Roudkenar et al., 2018). In fact, MSCs can secrete both angiogenic and anti-angiogenic factors in response to signals from microenvironment. For example, MSCs respond to TGFα/EGF receptor by increasing VEGF production (De Luca et al., 2011). On the other hand, VEGF signaling pathway is defective in TNFR2 KO mice (Luo et al., 2006), and a correlation between TNFR2 expression by MSCs and NO production, that directly induces VEGF, has been recently established (Beldi et al., 2020a). Previously, it has been found that VEGF production by TNF-α-primed human bone marrow MSCs was TNFR2 dependent (Crisostomo et al., 2008; Zhang A. et al., 2010).

As above mentioned, CXCL12/CXCR4 signaling pathway is important for *in vivo* MSC homing to injured sites but also increase VEGF expression, thus contributing to neoangiogenesis. Accordingly, CXCL12-secreting MSCs have improved wound healing, dermal fibroblast migration and new blood vessel formation (Nakamura et al., 2013), whereas CXCR4-overexpressing MSCs improved the outcome of myocardial infarction by increasing cell engraftment and angiogenesis and reducing myocardial remodeling (Huang et al., 2010; Zhang D. et al., 2010). Obviously, effects mediated by VEGF-overexpressing MSCs are related to the potent pro-angiogenic capacity of this molecule that improves the blood flow and the heart function in preclinical assays of critical limb ischemia (Beegle et al., 2016) and myocardial infarction (Zhu et al., 2012), respectively.

MSC overexpressing genes involved in osteogenesis, particularly BMP2 have been repeatedly tested in several types of bone defects, improving the bone healing (Chang et al., 2003, 2004, 2010; Tsuchida et al., 2003; Jiang et al., 2009; Zhao et al., 2010). Sometimes, BMP2 and VEGF overexpression have been combined. In these cases, VEGF promotes blood vessel neoformation that favors BMP2-mediated osteogenesis (Lin et al., 2010, 2011, 2012, 2015; Fu et al., 2015).

Engineered MSCs have been also used as anti-tumor therapeutic agents alone or in conjunction with chemotherapeutic drugs (Pawitan et al., 2020). Three strategies have been used: (i) to insert suicide genes that transform non-toxic pro-drugs into cytotoxic molecules; (ii) to use MSCs as vehicles to transport cytokines for enhancing *in vivo* anti-tumoral immunity or (iii) as agents to kill directly the tumor cells. Several genes encoding for “suicide proteins” have been used in anti-tumoral therapies, including cytosine deaminase, thymidilate kinase from either herpex simple or SV40 viruses, and cytochrome P450 reductase. In general, MSCs are resistant to these agents, particularly to alkylating agents, although evidence on the effects mediated by nucleoside analogs is scarce. IL-12 (Gao et al., 2010), IFNγ (Seo et al., 2011), and TNF-α (Tyciakova et al., 2015) have been overexpressed in engineered MSCs to enhance anti-tumoral immune responses. Another tested strategy is based on the use of MSCs to deliver pro-apoptotic agents to tumor cells. The most frequently used is the overexpression of TNF-related apoptosis inducing ligand (TRAIL), a transmembrane protein that binds to death domain-containing receptors that selectively trigger apoptotic of cancer cells (Luetzkendorf et al., 2010). Other approaches include engineered MSCs to release different anti-angiogenic factors (Zheng et al., 2012) or oncolytic viruses (Yong et al., 2009). In general, most studies using these strategies work quite well in preclinical models but their efficiency in human patients has been very limited.

Modifications of the culture protocols also change the MSC functionality and would be considered in this section. MSCs in three-dimensional culture conditions (3D) have shown an upregulated expression of TSG6, SCT1, LIF, IL24, TRAIL, and CXCR4 (Potapova et al., 2008). MSC spheroids generate reduced levels of some pro-inflammatory molecules such as TNF-α, IL-1β, CXCL12, MIP-2, and PGE_2_, and stimulate their pro-angiogenic activity, increasing their anti-fibrotic properties (Xu et al., 2016). It is important to remark that 3D spheroid cultures create a microenvironment where inner cell layers are exposed to lower levels of oxygen and nutrients generating a hypoxic environment (Cesarz and Tamama, 2016) that importantly affects the MSC biology (see below).

Furthermore, the nanoparticle use is being analyzed to enhance cell therapeutic efficacy. Engineering strategies that associate nanoparticles with MSC membranes have improved their homing ability, tumor tropism and attraction to inflammatory tissues (Wang et al., 2020). MSC-derived cell membrane coated nanoparticles have proven to be a useful biomimetic strategy to design therapeutic devices that have shown great potential in diagnostic and therapeutic applications. Among them, we can highlight the administration of drugs, immune modulation, vaccination and detoxification (Narain et al., 2017).

Hypoxia controls the MSC biology as well and it has been a target for improving their capabilities. In general, MSCs are cultured in normoxia, around 21% oxygen, but the optimal oxygen concentration can vary among tissues *in vivo*. The MSC niche is hypoxic, around 5% oxygen, compared to highly perfused organs. A low level of oxygen during the *in vitro* culture would make available a positive environment for MSCs to simulate their physiologic conditions. Thus, hypoxia could contribute to maintain the stemness and the proliferative capacities of MSCs during the *in vitro* culture. Choi J. R. et al. (2014) reported that adipose tissue-derived MSCs cultured in 2% oxygen tension maintained their stemness capacity, increased proliferation rate and enhanced their chondrogenic differentiation compared to MSCs cultured in normoxia with 21% oxygen. Indeed, numerous studies confirm hypoxia as a preconditioning factor of MSCs that induces increased production of pro-angiogenic factor (Liu et al., 2015), as well as anti-oxidative and anti-apoptotic effects in healthy and pathological conditions (Zhang W. et al., 2014).

These results were related with an increase in the expression of hypoxia-induced factor-1α (HIF-1α) under hypoxia (Choi J. R. et al., 2014). HIF-1α activation in MSCs cultured in hypoxia conditions induces increased expression of neovascularization promoters such as VEGF and angiotensin (Imtiyaz and Simon, 2010; Ahluwalia and Tarnawski, 2012). Roemeling-van Rhijn et al. showed that immunosuppressive properties of adipose tissue derived-MSCs were maintained under hypoxic conditions. The oxygen level had no effect on the proliferation of adipose tissue derived-MSCs and colony forming unit efficiency was similar under 1 and 20% oxygen. Also, they did not observe cell toxicity neither changes in the immunophenotype, except a downregulation in the expression of CD105 (Roemeling-van Rhijn et al., 2013). Martinez et al. transduced human dental pulp MSCs with a lentiviral vector codifying for HIF-1α. Compared to unmodified MSCs, HIF-1α-MSCs showed the same capacity to inhibit T cell activation, but HIF-1α-MSCs were able to impair DC differentiation more efficiently. As well, HIF-1α-MSCs induced higher attraction of monocytes, exhibited greater resistance to NK cell-mediated lysis and also exhibited a pro-angiogenic profile due to an increased expression of the chemokines CXCL12 and CCL5 and a complete loss of CXCL10 transcription (Martinez et al., 2017). Schive et al. investigated the *in vivo* therapeutic potential of hypoxic-cultured MSCs in a mouse model of streptozotocin-induced insulitis and hyperglycemia compared to MSCs cultured in normoxic conditions. Either hypoxic-cultured or normoxic-cultured MSCs were injected into this mouse model. Both groups of animals had higher pancreas insulin content compared to untreated control group, but the hypoxic-cultured MSC group had lower fasting blood glucose and improved oral glucose tolerance compared to untreated mice. The authors concluded that hypoxic preconditioning potentiates MSCs ability to protect against hyperglycemia *in vivo* (Schive et al., 2017).

Alternatively, 3D MSC cultures based on the different conditions of cultures vs. those of two-dimensional (2D) cultures constitute another way for improving the biological and therapeutic properties of MSCs. In fact, 3D MSC cultures reflect better the natural physiological environment than the 2D cultures. Thus, the use of 3D MSC cultures could mimic better the physiologic state of MSCs in their specific resident tissues and influence their paracrine mechanisms. The 3D MSC cultures are based on MSC spheroids encapsulated with various types of scaffolds such as hydrogels, polymers, hydrophilic glass fibers and electrospun silk fibroin meshes. Other approaches that are not based on the use of scaffolds include magnetic levitation, hanging drop microplates or ultralow attachment spheroid microplates (Langhans, 2018; Millan-Rivero et al., 2019; Sankar et al., 2019). The spheroid 3D cultures create a microenvironment in which inner layers are exposed to lower levels of oxygen and nutrients, resembling to a hypoxic environment that affects notably the MSC behavior. Compared to 2D cultures, 3D MSC cultures have shown an augmented secretion of molecules with paracrine function (i.e., cytokines, chemokines, and growth factors), better anti-oxidative and anti-apoptotic functions and higher production of extracellular matrix components (Cushing and Anseth, 2007; Sun et al., 2018; Mukherjee et al., 2020), as well as improved therapeutic effects in some preclinical models such as corneal or skin wound healing (Carter et al., 2019; Millan-Rivero et al., 2019).

This accumulating preclinical evidence on the promising potential of MSC-based cell therapy in the treatment of multiple diseases has allowed its translation to the clinical practice, having been launched to date more than 1,000 clinical trials. However, clinical trials based on the use of engineered MSCs are still very scarce, and only a few studies, mainly phase I and phase I/II, have been implemented to evaluate “MSCs-2.0” safety and efficacy in a variety of pathologic conditions summarized in Table 1, and their results are eagerly awaited.

**TABLE 1:** Clinical trials involving engineered MSCs in Clinicaltrials.gov website.

Study title	MSC source	Modification	Pathology	Phase	Identifier
MV-NIS infected MSCs for treating patients with recurrent ovarian, primary peritoneal or fallopian tube cancer	Adipose tissue	MSCs transduced with Edmonston’s strain measles virus (MV) genetically engineered to produce sodium iodine symporter (NIS)	Recurrent ovarian, primary peritoneal or fallopian tube carcinoma or adenocarcinoma	Phase I/II	NCT02068794
Genetically Modified MSC Therapy Against Head and Neck Cancer (Gx-051)	N/A (Gx-051)	MSCs expressing modified interleukin-12 (MSCs/IL-12M)	Head and neck neoplasm	Phase I	NCT02079324
Osteogenic effects in human MSCs enhanced by Wnt signaling	Bone marrow	Viral administration of Wnt3a-transduced MSCs with hydroxyapatite nanoparticles	Osteoarthritis	Observational	NCT01323894
Efficacy and safety of allogeneic MSCs of bone marrow, cultured under hypoxia in the treatment of patients with severe pulmonary emphysema	Bone marrow	MSCs cultured under hypoxic conditions	Severe pulmonary emphysema	Phase I/II	NCT01849159
A single dose of BRTX-100 for patients with chronic lumbar disc disease	Bone marrow (BRTX-100)	Hypoxic-cultured bone marrow mononuclear cells highly enriched in MSCs from autologous bone marrow with autologous platelet lysate	Chronic lumbar disc disease	Phase II	NCT04042844
Intravenous infusion of fucosylated bone marrow MSCs in patients with osteoporosis	Bone marrow	Enzymatic exofucosylation by fucosyltransferase VIII and GDP-fucose treatment	Osteoporosis with low impact bone fractures	Phase I	NCT02566655

*N/A, data not available. Fast track designation of commercial cell therapy products is also indicated.*

On the other hand, in the last years, several studies have emphasized the presumptive relevance of the MSC secretome as a better way of treatment than the own cells for their clinical application (Poltavtseva et al., 2019), although reported results are frequently contradictory and the clinical assays using total secretome or extracellular vesicles (EVs) are limited. On the other hand, despite the difficulties for a conclusive definition of their phenotype, content and physiological function, EVs present some benefits, such as low immunogenicity, stability during extended storage and protection of their content (Kusuma et al., 2017). The term “secretome” includes diverse soluble molecules, such as growth factors, cytokines, immunomodulatory molecules and the named EVs (Kusuma et al., 2017). EVs are a heterogeneous population of lipid-bilayer vesicles that contain biologically active biomolecules such as lipids, proteins, single-stranded DNA and different types of RNAs (Bister et al., 2020; Watanabe et al., 2021). They include small exosomes (40–120 nm) originated from multivesicular bodies of the endosomal compartment that are secreted by exocytosis, and larger microvesicles (200–1,000 nm) that bud directly from the plasma membrane (Kusuma et al., 2017).

Moreover, MSCs derived from different sources produce some specific factors: adipose tissue-derived MSCs secrete more IGF-1, VEGF, and IL-8 than those from the bone marrow, whereas MSCs from the umbilical cord Wharton’s jelly secrete the highest amounts of immunomodulatory molecules such as IL-6, IL-7, IL-10, PDGF-A, and TGFβ2. On the contrary, adipose tissue-derived MSCs produce more extracellular matrix components such as collagen-1 and -2, and metalloproteinases (Amable et al., 2014), and a common group of secreted molecules including chemokines (CCL2 and CCL5), growth factors (bFGF and IGF-1), cytokines (IL-6 and TGFβ) and others (TNFR-I) (Wang et al., 2019). However, not only soluble factors can be secreted by MSCs. Remarkably, mitochondria can be transferred between cells via tunneling nanotubes, cell fusion or contained into secreted EVs (Torralba et al., 2016; Morrison et al., 2017). Therefore, the secretome recapitulates many of the properties described for MSC themselves (Ferreira et al., 2018), including immunomodulation (Teng et al., 2015), inhibition of both apoptosis and fibrosis (Li et al., 2013; Teixeira et al., 2015), induction of vascularization (Teng et al., 2015) and promotion of tissue remodeling and cell recruitment (Chen et al., 2014). Furthermore, EVs derived from MSCs activated or not with IFNγ exhibit distinct capacities. Both EVs reduce the frequency of CD14^+^CD16^+^ inflammatory monocytes, but those derived from IFNγ-treated MSCs also promote anti-inflammatory PD-L1 expressing monocytes (Goncalves et al., 2017).

Although the number of clinical trials using MSC-derived EVs for the treatment of different pathologies is still very limited (see Table 2) and their conclusions unpublished, numerous preclinical studies support their functional capabilities. Different lung injuries improve after treatment with MSC-derived EVs. In acute respiratory distress syndrome (ARDS) models, the administration of MSC-derived CD44^+^ EVs reduced the lung injury (Morrison et al., 2017). Remarkably, EV-mediated mitochondrial transfer induces a highly phagocytic and an anti-inflammatory macrophage phenotype (Morrison et al., 2017). In addition, MSC-derived exosomes remodel vascular network and diminish the hypoxia pulmonary hypertension in rodent models (Weiss et al., 2019). Systemically injected EVs have been shown to reduce both the collagen deposits and the inflammatory infiltrates in a murine model of silica-induced lung fibrosis (Choi M. et al., 2014).

**TABLE 2:** Clinical trials involving MSC-derived EVs in Clinicaltrials.gov website.

Study title	MSC-ECVs source	Pathology	Phase	Identifier
Exosomes of MSCs for multiple organ dysfunction syndrome after surgical repair of acute type A aortic dissection	N/A	Surgical repair of acute type A aortic dissection	Not applicable	NCT04356300
MSC-exos promote healing of macular holes	Umbilical cord	Large and refractory macular holes	Phase I	NCT03437759
Effect of UMSCs derived exosomes on dry eye in patients with cGvHD	Umbilical cord	Dry eye symptoms in chronic GvHD	Phase I/II	NCT04213248
Safety and efficacy evaluation of allogeneic adipose MSC-exos in patients with Alzheimer’s disease	Adipose tissue	Mild/moderate dementia associated to Alzheimer’s disease	Phase I/II	NCT04388982
Effect of microvesicles and exosomes therapy on B-cell mass in type I diabetes mellitus	Umbilical cord blood	Type 1 diabetes mellitus	Phase II/III	NCT02138331
MSC-EVs in dystrophic epidermolysis bullosa	Bone marrow (AGLE-102)	Dystrophic epidermolysis bullosa	Phase I/II	NCT04173650
Expanded access protocol on bone marrow MSCs derived extracellular vesicle infusion treatment for patients with COVID-19 associated ARDS	Bone marrow (ExoFlo^1^”]	COVID-19 associated acute respiratory distress syndrome	Phase II	NCT04657458 NCT04493242
Clinical study of mesenchymal stem cell exosomes nebulizer for the treatment of ARDS	N/A	COVID-19 associated acute respiratory distress syndrome	Phase I/II	NCT04602104
Pilot clinical study on inhalation of MSC exosomes treating severe novel coronavirus pneumonia	Adipose tissue	COVID-19 pneumonia	Phase I	NCT04276987
Effects of ASC secretome on human osteochondral explants	Adipose tissue	Osteoarthritis and/or articular regeneration	Observational	NCT04223622
iExosomes in treating participants with metastatic pancreas cancer with KrasG12D mutation	N/A	Metastatic pancreatic ductal adenocarcinoma	Phase I	NCT03608631
Allogeneic MSC derived exosome in patients with acute ischemic stroke	N/A	Acute ischemic stroke	Phase I/II	NCT03384433

*N/A, data not available. Fast track designation of commercial cell therapy products is also indicated.*

The effects of MSC-derived EVs have been tested in other models of tissue fibrosis: treatment with EVs enriched in miRNA-let7c, a model of renal fibrosis, induced a downregulated expression of collagen IV, metalloproteinase-9, TGFβ1 and its receptor (Wang et al., 2016). Also, ECV enriched in miRNA-125b, that target Shh signaling activated in liver fibrosis, rescues liver progenitor cell expansion and stellate cell activation (Hyun et al., 2015). MSC-EVs containing miRNA22 improve cardiomyocyte survival in a murine model of myocardial ischemia-reperfusion (Arslan et al., 2013; Feng et al., 2014).

Interestingly, CD69^–/–^ mice, which produce less exosomes, have shown significant reduced bone junctions. This problem can be recovered after injection of EVs isolated from MSC conditioned media, a process presumably mediated by RNAs (Furuta et al., 2016). EVs from embryonic MSCs promote cartilage regeneration in a rat osteochondral defect model by increasing both neoformation of tissue and extracellular matrix components (Zhang et al., 2016), whereas exposure of MSC-derived EVs increases stem cell engraftment in irradiated bone marrow (Wen et al., 2016). Effects of MSC-derived EVs on the immune system reflect their origins and tend to show immunosuppressive properties. MSC-derived EVs containing miRNA inhibit macrophage activation by controlling NF-κB activation and induce changes in the profile of expression of several immune molecules, including IL-1β, COX-2, IL-10, TNF-α, MyD88, TLR-1, -4, -5, -7-9, IRAK1, and TRAF6 (Phinney et al., 2015). On the other hand, EVs from MSCs obtained from bone marrow increase the IL-10 production and the proliferation of Treg cells in PBMNC cultures stimulated through CD3/CD28 (Del Fattore et al., 2015; Dal Collo et al., 2020).

## Comments and Conclusion

**FIGURE 1:**
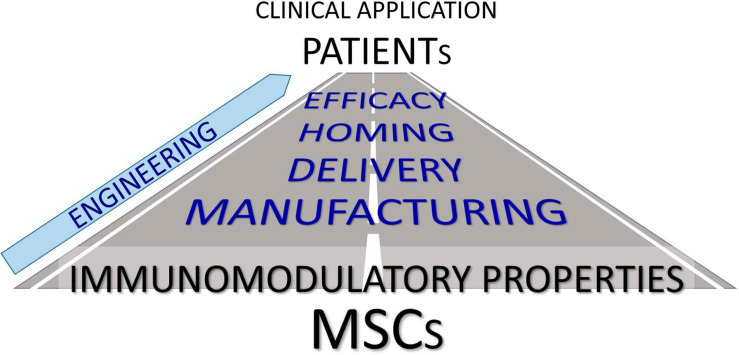
Next steps to improve the immunomodulatory properties of MSCs to treat patients efficiently.

We have reviewed the current state of the art of the biology of MSCs with special emphasis to the advances that might improve their therapeutic efficiency. A summary of the topics discussed is shown in Figure 1. Firstly, we reported data on the phenotypical and functional characteristics of MSCs highlighting the difficulties to get specific markers that would allow to isolate enriched, homogeneous MSC subpopulations and to identify one or a few molecules masters for governing their properties.

MSC were used for the first time as cellular pharmaceutical agents in humans in 1995 (Lazarus et al., 1995). After several years utilizing MSCs as therapeutical agents, numerous questions on their behavior remain unsolved, including the heterogeneity of the MSC populations in the final product, the adequate conditions to activate *in vivo* their immunomodulatory capabilities, the consequences of the banking procedures, the best route for their delivery, the use of allogeneic vs. autologous cells, the problems to reach the target host tissues, their response to stressful conditions, specially hypoxia, or the real therapeutic relevance of products secreted by MSCs, such as the soluble fraction of their secretome or EVs.

Accordingly, we have summarized recent published results on these issues in an attempt to provide new approaches for a better clinical application of the named “MSCs-2.0.” On the other hand, it is urgent a universal standardization of the protocols for manufacturing MSCs such as MSC culture and banking conditions, and also the route of cell delivery, the optimal dosage and the best way to use allogeneic vs. autologous MSCs. The available results on the effects of cryopreservation on MSC biology are contradictory and, although the use of allogeneic MSCs exhibit evident advantages in cell therapy, it is important to recognize their unquestionable immunogenicity, although immune responses elicited by allogeneic MSCs appear to be lower or less aggressive than autologous ones, presumably because a balance between immunogenicity and release of immunosuppressive factors is established in these circumstances.

Differences between MSC homing in preclinical models and humans must be conclusively clarified as well as the mechanisms governing the MSC homing into the target tissues by their relevance for a definitive establishment of the best route for MSC delivery, according to the disease to be treated. On the other hand, because MSC-mediated thromboembolism limits the MSC migration to the target tissues, this physiological condition, would be carefully evaluated before the systemic infusion of MSCs. Some simple, although transient, chemical manipulations of MSCs for improving their homing are highly promising but require technical optimization and a better knowledge on their consequences for the MSC biology. Also, usage of MSCs on scaffolds of diverse origin is complex and needs further research and improvement.

As indicated, to manipulate the whole factors known to affect MSC behavior is highly improbable since the therapeutic application of MSCs in a concrete disease requires the strengthening of the action of one or few discrete molecules. As summarized herein, genetic procedures have been attempted in the last years. However, this approach has the same problems than those reported in gene therapies applying other cell types. On the other hand, it is obvious that MSC engineering is a robust technology extensively tested in numerous experimental models but the translation of these results to the clinical practice need more time and research to be successful.

Apart from gene overexpression procedures, MSCs can be engineered changing the culture conditions by using 3D cultures or hypoxic conditions, or adhering distinct types of nanoparticles to the MSC membrane. Hypoxia has been frequently used as a preconditioning factor that favors MSC stemness and proliferation, and exhibit pro-angiogenic, anti-oxidant and anti-apoptotic effects.

EVs obtained from MSC secretome have provided in the last years numerous although frequently contradictory results but few effective clinical trials. Unfortunately, their heterogeneous condition, the lack of specific markers for establishing their true nature and, in general, the absence of information on the mechanisms controlling their effects, make difficult their therapeutic use, although increased numbers of clinical trials are being currently reported. It is therefore important to establish conclusively their real clinical value.

In summary, many of these research fields that try to improve MSC efficiency are ongoing with promising preclinical results, although the translation of their findings to the clinical practice seems to be yet remote.

## Author Contributions

DG-B, JS, MG-A, RY, RH-S, AC, MF-G, MH-R, ÓQ-B, JB, DG-O, JM, and AZ contributed to intellectual discussion of the findings. DG-B, JS, MG-A, RY, and AZ supervised the writing and drafting the manuscript. All the authors revised and approved the final version of the manuscript.

## Conflict of Interest

The authors declare that the research was conducted in the absence of any commercial or financial relationships that could be construed as a potential conflict of interest.
